# Prevalence, correlates, and trends of intimate partner violence against women in Pakistan: Results from Pakistan Demographic and Health surveys 2012–13 and 2017–18

**DOI:** 10.1371/journal.pone.0298681

**Published:** 2024-03-21

**Authors:** Masood Ali Shaikh

**Affiliations:** Independent Researcher, Karachi, Pakistan; COMSATS University Islamabad - Lahore Campus, PAKISTAN

## Abstract

**Background:**

Intimate partner violence (IPV) is a global public health problem. The objectives of this study were to analyze the prevalence and correlates of IPV perpetrated by men against women from the recent nationally representative Pakistan Demographic and Health Survey (PDHS) 2017–18, and to analyze levels and trends of IPV perpetrated by current/former husbands from PDHS conducted in 2012–13, in the four provinces and the capital city.

**Methods:**

Association of having ever experienced IPV, defined as either emotional, physical and/or sexual violence, by ever married women aged 15–49, with 12 explanatory socio-demographic, attitudinal, and experiences were analyzed using simple and multiple logistic regression models.

**Results:**

The prevalence of having ever experienced IPV was 33.48% (95% CI: 30.76–36.32). In the final multivariable model, number of living children, having knowledge of parental physical IPV, husband’s use of alcohol, and marital control were statistically significantly associated with IPV. Proportions and trend analysis of emotional and physical IPV between the PDHS 2017–18 and PDHS 2012–13, showed that in general, rural areas of provinces reported higher prevalence of emotional and physical IPV, compared with urban areas, and in general, emotional, and physical IPV prevalence declined from PDHS2012-13 to PDHS2017-18.

**Conclusions:**

The prevalence of having experienced physical and/or sexual intimate partner violence in Pakistan was lower than the prevalence for the WHO Eastern Mediterranean region. However, IPV burden at the provincial urban-rural residency status underscore the need for location specific strategies to effectively address IPV in Pakistan.

## 1. Introduction

Violence against women is a health and human rights problem globally. The United Nations Sustainable Development Goals (SDG), goal 5 strives to “achieve gender equality and empower all women and girls” [1]. The target 5.2 endeavors to “eliminate all forms of violence against all women and girls in the public and private spheres, including trafficking and sexual and other types of exploitation” [1]. Based on 2018 estimates, the World Health Organization (WHO) reported that globally, 27% of ever married/ partnered women aged 15–49 years experienced physical and/or sexual violence at least once in their lifetime [2]. While in the WHO Eastern Mediterranean Region, the lifetime prevalence in this demographic group was 31% [2]. The lowest such prevalence was reported from the WHO Western Pacific and European regions with 20% and 21%, respectively. While the highest prevalence was reported from the WHO regions of South-East Asia and African regions, with 33% each [2].

A recent systematic literature review based on 2000–2018 WHO Global Database reported 27% lifetime prevalence of physical and/or sexual intimate partner violence (IPV) in the ever-partnered women aged 15–49 years, pretreated by male partner. This review reported higher prevalence in low-income countries compared to high-income countries [3]. A meta-analysis based on 44 nationally representative surveys conducted in the sub-Saharan Africa (SSA) underscored the role of low education and rural residency status as high-risk attributes for IPV [4]. Another study based on 25 SSA countries highlighted the role of “inadequate living conditions” in terms of unimproved water, sanitation facilities, insufficient space, and unfinished materials in the house as a strong associative factor with IPV [5]. Moreover, this study also reiterated the association of IPV with rural residency status and low education. While another meta-analysis reported the association of IPV with infertility in women [6]. Other studies have also reiterated the role of lower socioeconomic status being strongly and consistently associated with higher levels of IPV, in several European countries [7,8].

Part of south Asia and WHO Eastern Mediterranean Region, Pakistan is the fifth most populous country in the world [9]. There have been only two nationally representative surveys on the intimate partner violence (IPV) in Pakistan and both were conducted by the ‘DHS Program’ [10]. Over 400 Demographic and Health Surveys (DHS) have been conducted in more than 90 countries; in Pakistan four standard DHS surveys were done, but only the last two included the ‘Domestic Violence’ module, that includes questions on IPV i.e. the Pakistan Demographic Health Survey 2012–13 (PDHS2012-13) and the Pakistan Demographic and Health Survey 2017–18 (PDHS2017-18).

Several studies have been published using the PHDS2012-13 and one study using the PHDS2017-18 data on the correlates of IPV’s. In Pakistan, IPV has been reported to be associated with increased risk for child and infant mortality, low birth weights and lower immunization in children [11]; husbands use of alcohol, lower educational attainment by women and their husbands [12,13]; having knowledge of one’s father physically beat her mother and women’s attitudinal acceptance of IPV [13,14]; lower economic status [15]; women’s acceptance of IPV [16]; rural residency status and lower number of antenatal care visits [17]; and younger age of women [18]. While one study using both i.e. PHDS2012-13 and PHDS2017-18 data on IPV correlates reported association of IPV with poor pregnancy outcomes in terms of number of lost pregnancies, terminated pregnancies, and reduced probability of delivery in a health facility in both surveys [19].

Broadly, the correlates associated with IPV tend to underscore low educational attainment by both partners, low socioeconomic status, use of alcohol by male partner, physical power differences, attitudinal acceptance of violence in the context of intimate relationships by women, rural residency status; all conspiring against the backdrop of deeply entrenched patriarchal cultures, traditional family values emphasizing harmony and ideological pressures to preserve marriages, force women to remain imprisoned in abusive relationships [3,4,14,20–23].

IPV, like any other public health concerns, manifests differential distribution at the subnational levels [24], that could potentially be masked when studying prevalence at the national level alone, in addition to differentials in terms of time. Hence, it is imperative to study geographical and temporal inequalities so as to identify subnational and temporal heterogeneities of IPV, to facilitate geographically tailored controlling and preventive interventions.

Studying the IPV trends and their statistical significance reveals its scope and evolution, pinpointing genuine patterns through rigorous analysis. This data-driven approach reveals the true strength and direction of trends across regions and ethnicities, empowering pursuit of targeted interventions precisely where they’re most needed. This crucial analysis provides robust evidence to refine strategies, monitor societal influences, potentially fuels advocacy for effective policies and increased resources. Thus, paving the way for a future free from IPV where action is guided by certainty, not chance.

The objectives of this study were two-fold: to analyze the prevalence and correlates of intimate partner violence (IPV) perpetrated by men against women using the most recent and nationally representative PDHS2017-18, and secondly to quantify the magnitude and trends of IPV perpetrated by current/former husbands using data from the PDHS2012-13 to PDHS2017-18 at the national, provincial, and provincial urban/rural levels, for the four major provinces as well as the capital city of Pakistan.

## 2. Materials and methods

### 2.1 Study area and data source

This secondary analysis was based on 2017–18 and 2012–13 cross-sectional, demographic and health surveys (PDHS) data from Pakistan. For the PDHS 2017–18, the data collection phase lasted from November 22, 2017, to April 30, 2018. Pakistan is administratively subdivided into 4 provinces (Punjab, Sindh, Khyber Pakhtunkhwa, and Balochistan), Islamabad Capital Territory (ICT), Federally Administered Tribal Areas (FATA), Azad Jammu Kashmir, and Gilgit Baltistan. The four provinces and ICT collectively account for over 97% of Pakistan’s population of 207 million, based on the census conducted in 2017 [25]. The PDHS surveys are representative at the national, provincial and ICT levels. Although the PDHS2017-18 is the fourth such survey, but the domestic violence module was used only in the 2012–13 and 2017–18 surveys.

Based on the 2017 census, the list of enumeration blocks (EB) was used as a sampling frame for the PDHS2017-18. Using the stratified two-stage sample design, with first stage entailing selection of clusters comprising of EBs drawn with probability proportional to size (PPS). There were in total 580 EBs (clusters) selected comprising of households, out of which survey was conducted in 561 clusters owing to security issues during the data collection phase. The second stage entailed, systematic sampling of households in which 28 households per EB/cluster were selected with PPS. For the ‘Domestic Violence’ module that includes questions on IPV, the PDHS2017-18 included ever-married women aged 15–49 years old. The domestic violence module was administered to one randomly selected eligible woman, in one-third of the sample households; after obtaining verbal informed consent.

The PDHS2017-18 was conducted by the National Institute of Population Studies, with technical support from Pakistan Bureau of Statistics and the ICF. The ethical approvals for the survey protocol were granted by the National Bioethics Committee of the Pakistan Health Research Council, and the Institutional Review Board of the ICF.

The approval for this secondary analysis was received from the DHS Program (www.dhsprogram.com) using the online request form, after registration. PDHS 2017–18 and 2012–13 datafiles were downloaded in the Stata program format from the DHS website. The PDHS2017-18 and PDHS2012-13 cumulatively selected 4,085 and 3,687 women for the domestic violence module, respectively. However, 131 women for PDHS2017-18 and 121 women for PDHS2012-13 could not be interviewed due to lack of privacy, interruptions during the interview, or inability to find the selected woman for interview despite repeated attempts. Details of both PDHSs pertaining to sampling design, survey methodology, generation of sampling weights, adjustments for non-response, and survey questionnaires are available in the country reports available for free download on the DHS website.

### 2.2. Study variables

Standardized domestic violence modules were used for the PDHSs entailing modified version of the Conflict Tactics Scale [26,27]. The determination of various types of IPV and their correlates have been described previously [28], a brief description is provided below.

### 2.3. Outcome variable

Intimate partner violence (IPV) was defined as a woman who reported having ever experienced any form of either emotional, physical, and/or sexual violence from either their current or former husband and coded as a dichotomous outcome variable. The emotional IPV questions entailed having ever experienced being humiliated, threatened to be harmed, insulted, or having made to feel bad. The physical IPV questions inquired about having ever experienced being pushed, shaken, or something thrown at, slapped, arm twisted, hair pulled, punched with a fist or something that hurt, kicked, dragged, strangled, burned, threatened with a knife, gun, or any other weapon. While sexual IPV questions pertained to ever having been physically forced into unwanted sex, physically forced into unwanted sexual acts, or physically forced to perform sexual acts that respondent did not want to. Affirmative answers to any of questions were dichotomously coded as present or absent.

### 2.4. Explanatory variables

Previous studies from Pakistan and numerous other countries have identified several IPV correlates [3–5,8,11–18,28–32]; in this study, twelve variables at the individual, husband/partner, and familial levels were analyzed for association with respondent having ever experienced intimate partner violence i.e. women’s’ age, women’s and her husband’s educational attainment, women’s occupation, wealth index of the household, residential status in terms of urban and rural, number of living children, participation in decision making, acceptance of IPV, alcohol use by husband, having knowledge of one’s father physically beating up one’s mother, and marital control. Details on the derivation of each explanatory variable has been reported previously [28]. Briefly, all variables were dichotomized with ‘don’t know’ responses coded as ‘no’. Any type of IPV acceptance, participation in any decision-making event, or any type of marital control exhibited by the husband, were coded as ‘present/yes’ if replied affirmatively.

Decision-making: The respondent holds the decision-making power for her healthcare, large household purchases, and family/relative visits, either independently or jointly with her husband. Acceptance of IPV: The respondent believes that beating is justified if wife goes out without telling her husband, neglects children, argues with husband, refuses to have sex with her husband, or burns food. Controlling behavior: The respondent experiences instances of controlling behavior from her husband, such as jealousy if she talks to other men, accusations of unfaithfulness, doesn’t permit her to meet her female friends, tries to limit contact with her family, or insists on knowing where she is. While age, wealth, number of living children, educational attainment, and occupation were coded as listed in the Table 1.

**Table 1:** Counts and proportions of study variables–Pakistan DHS 2017–18.

Variable	Unweighted count	Percentage
	(N = 4,085)	(Weighted)
***Outcome Variable***		
Intimate Partner Violence	No = 2,579	
(Emotional, physical, and/or sexual)	Yes = 1,505	33.48%
	Missing = 1	
*Emotional violence*	No = 2,842	
	Yes = 1,243	25.84%
*Physical violence*	No = 3,079	
	Yes = 1,004	22.93%
	Missing = 2	
*Sexual violence*	No = 3,900	
	Yes = 184	4.79%
	Missing = 1	
***Explanatory Variables***		
Age	15-19y = 146	4.31%
	20-24y = 555	17.05%
	25-29y = 797	20.66%
	30-34y = 816	19.53%
	35-39y = 797	16.20%
	40-44y = 512	10.99%
	45-49y = 462	11.27%
Respondent’s Education	No education = 2,087	49.56%
	Primary = 565	15.49%
	Secondary = 818	20.47%
	Higher = 615	14.48%
Husband’s Education		
	No education/Don’t know = 1,114	29.66%
	Primary = 538	16.29%
	Secondary = 1,348	32.89%
	Higher = 964	21.16%
	Missing = 1	
	* Not applicable = 120	
Occupation		
	Professional/Technical/Managerial/Clerical/Sales/Services = 265	5.14%
	Not working = 3,432	81.08%
	Agriculture (self-employed)/Skilled manual/Unskilled manual = 386	13.78%
	Missing = 2	
Wealth	Poorest = 794	17.52%
	Poorer = 943	19.82%
	Middle = 787	19.52%
	Richer = 747	20.52%
	Richest = 814	22.62%
Residence	Urban = 1,978	37.42%
	Rural = 2,107	62.58%
Children	0 = 469	13.65%
	1–2 = 1,188	32.23%
	3–4 = 1,308	30.56%
	5–14 = 1,120	23.56%
Decision making	Participated = 2,498	61.32%
	Not participated = 1,467	38.68%
	*Not applicable = 120	
Acceptance	Not justified/Don’t know = 2,245	59.23%
	Justified = 1,840	40.77%
Alcohol use	No = 3,914	96.42%
	Yes = 171	3.58%
Knowledge of parental IPV	No = 3,105	79.41%
	Yes = 978	20.59%
	Missing = 2	
Marital control	No/Don’t know = 2,788	71.94%
	Yes = 1,296	28.06%
	Missing = 1	

* Asked from currently married women only.

### 2.5. Statistical analysis

The STATA version 17.1 (StataCorp, 2021,Texas, USA) was used for all analysis while adjusting for complex sample design. Hypothesis testing including trends analysis was 2-tailed, with statistical significance set at 2-sided P < 0.05.

The analysis used 4-step process: in the first step unweighted counts, missing records, and cumulative weighted percentages were calculated for outcome and explanatory variables using PDHS2017-18. In the second step, simple binary logistic regression models were used to determine the statistical significance of every explanatory variable’s association with the outcome variable of having ever experienced IPV. The odds ratios, their statistical significance, and 95% confidence intervals were also calculated. In the third step, explanatory variables found to be statistically significantly associated with the IPV in the step two, were used in the binary multiple logistic regression model, and adjusted odds ratios, their statistical significance, and 95% confidence intervals were calculated. In the final step, the three types of IPV were calculated for PDHS2012-13, and as well as for PDHS2017-18 by province, disaggregated by urban/rural status in each province, and for the ICT region. Followed by comparison of trend between the two surveys: by merging both data files together and using year as a dichotomous variable in the binary simple logistic regression model, with IPV as explanatory variable.

## 3. Results

Cumulatively, 1,505 women reported one or more of the three types of spousal violence. Emotional, physical, and sexual violence was reported by 1,243, 1,004, and 184 women, respectively. While 135 women reported all three types of IPV (weighted proportion 3.44%, 95%CI: 2.55–4.64); 761 women reported physical and emotional violence (weighted proportion 15.92%, 95%CI: 14.02–18.03); 154 women reported physical and sexual violence (weighted proportion 4.03%, 95%CI: 3.05–5.32); and 146 women reported both emotional as well as sexual violence (weighted proportion 3.58%, 95%CI: 2.67–4.78). There were 1034 women who reported physical and/or sexual IPV (weighted proportion 23.68%, 95%CI: 21.23–26.32).

Table 1 shows the results of exploratory data analysis in terms of outcome and explanatory variables’ unweighted counts, and cumulative weighted percentages, in terms of providing indices for the entire sample of women who answered IPV questions; based on the 4,085 women aged 15–49 years. Who were either currently or formerly married and completed the IPV questions on the domestic violence module of PDHS-2017-18. The results pertain to spousal violence committed by the current husband or the most recent husband for those women who were either divorced, separated, or widowed. For 120 women, information on husband’s educational attainment, and decision making in the areas of healthcare seeking for self, large household purchases and visits to relatives, were not available as these questions were asked from only those women who were currently married–as opposed to formerly married–women.

The prevalence of having ever experienced emotional, physical, and/or sexual spousal violence perpetrated by either current or most recent husband was 33.48% (95% CI: 30.76–36.32) in women aged 15–49 years. While emotional, physical, or sexual IPV were reported by 25.84% (95% CI: 23.47–28.35), 22.93% (95% CI: 20.54–25.52), and 4.79% (95% CI: 3.69–6.21) women respectively. The most common type of physical violence reported was ever having been slapped by husband (20.43% - 95% CI: 18.32–22.71); most common type of emotional violence reported was ever having been humiliated by husband (22.17% - 95% CI: 19.92–24.59); while the most common type of sexual violence reported was ever having been physically forced into unwanted sex by husband (4.53% - 95% CI: 3.45–5.93).

Cumulatively among the survey respondents, 42.02% women were under the age of 30 years; half (49.56% had no formal education; 29.66% women’s husbands had no education or women did not know the level of their husband’s educational level; 81.08% did not work; 37.34% fell in the wealth index quintiles comprising of poorest and poorer; 62.58% were rural dwellers; 67.79% had 1–4 children; 61.32% made major decisions either alone or jointly with their husband; 59.23% did not believe violence was acceptable; husband’s use of alcohol was reported by 3.58%; 79.41% did not know whether know whether their father had ever physically beaten their mother; and marital control was exhibited by 28.06% of women’s husband.

Table 2 shows the results of simple and multivariable logistic regression models in terms of crude Odds Ratios (OR), adjusted odds ratios (aOR), their statistical significance, and the associated 95% confidence intervals (CI). Out of the twelve explanatory variables examined in the bivariate analysis, nine were found to be statistically significantly associated with having ever experienced any type of intimate partner violence. All these nine explanatory variables i.e. educational attainment, occupation, partner’s educational attainment, wealth status, number of living children, acceptance of IPV, husband/partner’s use of alcohol, having knowledge of parental physical IPV, marital control, and participation in decision making were added in the multivariable logistic regression model. The results in this table show that four explanatory variables were statistically significantly associated with the IPV experience in the multivariable model i.e. number of living children, having knowledge of parental physical IPV, husband use of alcohol, and marital control.

**Table 2:** Crude odds ratios and adjusted odds ratios for all statistically significant associations between intimate partner violence and the selected variables—Pakistan DHS 2017–18.

Explanatory variable	Unadjusted OR	p-value	95% CI	Adjusted OR	p-value	95% CI
**Age**						
15–19	Reference			Not Applicable		
20–24	0.75	0.333	0.43–1.34			
25–29	1.10	0.738	0.62–1.97			
30–34	1.37	0.295	0.76–2.45			
35–39	1.20	0.540	0.67–2.13			
40–44	1.21	0.498	0.69–2.13			
45–49	1.35	0.320	0.75–2.44			
**Education (Respondent/Women)**						
No Education	Reference			Reference		
Primary	1.15	0.379	0.84–1.56	1.07	0.706	0.74–1.57
Secondary	0.68	0.018	0.50–0.94	0.94	0.758	0.62–1.42
Higher	0.45	<0.001	0.30–0.68	0.83	0.456	0.52–1.35
**Education (Husband)**						
No Education	Reference			Reference		
Primary	1.22	0.257	0.87–1.72	1.44	0.056	1.00–2.09
Secondary	0.81	0.169	0.61–1.09	1.20	0.292	0.85–1.70
Higher	0.56	0.002	0.39–0.80	1.00	0.984	0.66–1.51
**Occupation**						
Professional, Technical, Managerial, Clerical, Sales, Services	Reference			Not applicable		
Not working	1.05	0.803	0.70–1.58			
Agriculture, Self-employed, Skilled manual, Unskilled manual	1.25	0.418	0.73–2.12			
**Wealth**						
Poorest	Reference			Reference		
Poorer	1.37	0.052	1.00–1.87	1.32	0.119	0.93–1.87
Middle	1.05	0.792	0.72–1.54	1.11	0.640	0.71–1.74
Richer	0.85	0.397	0.59–1.23	1.14	0.586	0.71–1.82
Richest	0.61	0.023	0.40–0.93	0.93	0.777	0.54–1.59
						
**Residence**						
Urban	Reference			Not Applicable		
Rural	1.28	0.053	1.00–1.65			
**Children**						
No children	Reference			Reference		
1–2 children	2.25	<0.001	1.53–3.32	2.79	<0.001	1.78–4.36
3–4 children	2.61	<0.001	1.79–3.78	3.41	<0.001	2.18–5.34
5–12 children	3.99	<0.001	2.76–5.77	4.51	<0.001	2.79–7.28
**Decision making**						
Did not participate	Reference			Reference		
Participated	0.71	0.004	0.57–0.90	0.82	0.170	0.61–1.09
**Acceptance of IPV**						
Not justified	Reference			Reference		
Justified	1.59	<0.001	1.25–2.02	0.85	0.233	0.64–1.11
**Alcohol use by husband**						
Does not use alcohol	Reference			Reference		
Uses alcohol	7.96	<0.001	4.15–15.26	4.72	<0.001	2.39–9.30
**Knowledge of parental IPV**						
No	Reference			Reference		
Yes	3.68	<0.001	2.86–4.73	3.10	<0.001	2.36–4.06
**Marital Control by husband**						
No	Reference			Reference		
Yes	6.15	<0.001	4.75–7.98	5.24	<0.001	4.07–6.73

OR = Odds Ratio.

In the final multivariable logistic regression model, compared to women with no children, women with 1–2, 3–4, and 5–14 children experienced an increase of IPV with adjusted odds ratios (aOR) of 2.79 (95% CI: 1.78–4.36), 3.41 (95% CI: 2.18–5.34), and 4.51 (95% CI: 2.79–7.28), respectively. Odds were 3.10 times (95% CI: 2.36–4.06) higher for women who had knowledge of parental physical IPV, compared to women who did not know whether their father ever beat their mother. Odds of IPV were 4.72 times (2.39–9.30) higher in women whose husband or partner used alcohol, compared to those women whose husband or partner did not use alcohol. While the odds of IPV were 5.24 times (95% CI: 4.07–6.73) higher in women whose husbands exhibited marital control, compared to those women whose husbands did not.

Proportions and trend analysis of emotional and physical IPV between the PDHS 2017–18 and PDHS 2012–13, disaggregated by the four provinces and ICT individually, as well as by provincial urban/rural residency status, are shown in Table 3. Highest proportion of emotional IPV was reported from KPK, while lowest proportion was reported from Sindh in both surveys. For physical IPV, highest proportions were reported from KPK in the PDHS 2012–13, and from Balochistan in PDHS 2017–18, while lowest proportions were reported from Sindh in both surveys.

**Table 3:** Proportions and trend analyses for emotional and physical violence by province and provincial urban/rural disaggregation—Pakistan DHSs 2012–13 and 2017–18.

Type of violence	DHS 2012–13 Percentage	95% CI	DHS 2017–18 Percentage	95% CI	Trend Statistical Significance (p-value)
**Emotional Violence *(Rural & Urban Combined)***	** **		** **		** **
Punjab	34.92	31.07–38.98	23.33	20.30–26.65	<0.001
Sindh	14.43	11.68–17.69	12.94	10.06–16.50	0.505
KPK	47.27	42.29–52.30	48.25	39.46–57.14	0.850
Balochistan	42.93	32.28–54.27	29.84	23.74–36.76	0.041
ICT	33.88	25.20–43.80	25.68	18.38–34.66	0.190
**Physical Violence *(Rural & Urban Combined)***					
Punjab	23.15	20.01–26.62	21.25	17.94–24.98	0.438
Sindh	19.84	16.35–23.86	11.94	9.09–15.53	0.002
KPK	50.70	45.32–56.07	35.15	27.55–43.58	0.002
Balochistan	39.26	29.59–49.86	44.36	35.86–53.21	0.454
ICT	24.24	17.92–31.91	17.09	11.68–24.32	0.135
**Emotional Violence *(Rural)***					
Punjab Rural	33.03	27.30–39.31	21.31	17.74–25.37	0.001
Sindh Rural	12.17	9.10–16.09	13.62	9.24–19.63	0.633
KPK Rural	35.52	28.95–42.70	51.05	40.48–61.53	0.016
Balochistan Rural	36.43	21.94–53.89	29.78	21.97–38.98	0.458
**Emotional Violence *(Urban)***					
Punjab Urban	35.84	30.82–41.19	26.74	21.66–32.52	0.019
Sindh Urban	16.34	12.12–21.66	12.36	8.79–17.11	0.208
KPK Urban	49.52	43.77–55.30	35.97	26.78–46.33	0.023
Balochistan Urban	44.76	31.70–58.58	29.99	21.17–40.58	0.078
**Physical Violence *(Rural)***					
Punjab Rural	24.42	18.78–31.12	20.01	16.44–24.14	0.217
Sindh Rural	14.84	11.10–19.56	13.43	8.43–20.72	0.706
KPK Rural	39.21	30.02–49.24	37.57	28.43–47.68	0.809
Balochistan Rural	40.13	28.13–53.44	45.89	34.44–57.80	0.507
**Physical Violence *(Urban)***					
Punjab Urban	22.52	18.82–26.71	23.34	17.17–30.89	0.837
Sindh Urban	24.09	18.73–30.40	10.68	7.96–14.18	<0.001
KPK Urban	52.91	46.74–58.98	24.58	17.30–33.67	<0.001
Balochistan Urban	39.02	27.09–52.43	40.78	30.81–51.57	0.830

Regarding rural-urban disaggregation: highest proportion of emotional IPV was reported from rural Balochistan in PDHS 2012–13 and in rural KPK in PDHS 2017–18. Lowest proportions were reported from rural Sindh in both surveys. For urban areas, highest proportions of emotional IPV were reported from urban KPK in both surveys. While lowest proportions were reported from urban Sindh in both surveys. Regarding physical IPV, highest proportions of physical IPV were reported for rural Balochistan and lowest from rural Sindh in both surveys. For urban areas, highest proportion of physical IPV were reported from urban KPK in PDHS 2012–13, and urban Balochistan in PDHS 2017–18. While lowest proportions were reported from urban Punjab in PDHS 2012–13 and from urban Sindh in PDHS 2017–18.

Except for KPK, all other provinces and ICT reported declines in emotional violence from PDHS 2012–13 to PDHS 2017–18. However, this decline was statistically significant in Punjab and Balochistan. For physical IPV, apart from Balochistan, all provinces and ICT reported declines from PDHS 2012–13 to PDHS 2017–18; however, only Sindh and KPK were found to be statistically significant. Regarding rural-urban disaggregation: for emotional violence the rural Sindh and rural KPK reported increases while the other two provinces reported decline from 2012–13 to 2017–18; with this decline being statistically significant in rural Punjab and rise statistically significant in rural KPK. For urban areas, all provinces reported declines from 2012–13 to 2017–18 but statistically significant declines were only in urban Punjab and urban KPK. Regarding physical IPV, all rural areas reported declines from 2012–13 to 2017–18, except for rural Balochistan that reported rise; but none were statistically significant. For urban areas, urban Punjab and urban Balochistan reported rise in physical IPV from 2012–13 to 2017–18, that were not statistically significant. While urban Sindh and urban KPK reported statistically significant declines.

## 4. Discussion

The purpose of this study was to gain a better understanding of the correlates of violence against women in the context of intimate partner relationships using the most recent nationally, provincially, and capital city representative survey data i.e. PDHS2017-18, and to analyze the levels and trends of IPV compared with the previously representative survey data from the PDHS2012-13. By untangling real trends from chance fluctuations and comparing IPV across regions and ethnicities, these results illuminate where and how to build a future free from IPV, guided not by guesswork, but by the unwavering light of evidence. Based on the PDHS2017-18, one-third of women reported having ever experienced emotional, physical, and or sexual violence perpetrated by their current or former husband. The most common type of IPV reported was emotional violence, closely followed by physical violence. This pattern of emotional violence as the leading type of IPV followed by physical IPV is in contrast with other studies using DHS data that have reported physical IPV as the most common IPV type, including the neighboring country of Afghanistan [28–30]; however, emotional IPV was only 2.91 percent points higher than physical IPV prevalence. The prevalence of physical and/or sexual IPV in Pakistan was lower than the global and the WHO Eastern Mediterranean regional proportions.

Results of bivariate analyses of IPV correlates using the PDHS2017-18 data revealed that age, occupation, and residential status in terms of urban and rural residency were not statistically significant correlates of IPV. This is in contrast with previous report from Pakistan using PDHS 2012–13 data, where younger age of women was associated with increased IPV prevalence in Pakistan [18]. However, heterogenous relationship with age and IPV has been reported in other countries, with both younger and older age of women being associated with increased reporting of IPV [5,29–31]. The widespread nature of being without a job (80% of respondents) perhaps limited its usefulness as a correlate of IPV. However, lack of association between occupational status and IPV has also been reported in other studies [24,28,30]. The lack of statistical significance of the urban-rural residency status at the national level was a disparate finding from the PHDS2012-13 as well as a recent meta-analysis [4,17]. In general rural denizens tend to have lower educational attainment, economic status, and more pronounced patriarchal societies. Yet, this lack of association might reflect IPV pervasiveness, but such lack of association has also been previously reported [24,28]. While results of multivariable analyses showed the statistically significant associations of IPV with the number of living children; partner’s use of alcohol; having knowledge of IPV at home; and marital control. Compared with no living children, women with higher number of living children reported higher IPV in this study; a finding in consonance with other studies [30,31]. Association of number of children with IPV showed a clear gradient with increasing number of children being associated with increasing odds of IPV reporting. This could be explained by the fact that having more children perhaps results in more pressure on a husband to provide and care for children with resultant stress exhibiting as IPV; which wouldn’t be too anomalous in patriarchal societies. Use of alcohol by male partner is almost a universal finding in studies on IPV in heterosexual unions [28,31]; perhaps owing to poor judgement and limited impulse control in alcohol users. In addition to being a traumatic event, having knowledge of one’s father physically beat up one’s mother, could potentially result in internalizing such malevolent behavior as a norm in the context of marital union. Association between having such knowledge in women and higher IPV has been widely documented [14,28,30,31]. While marital control can be a prelude and precursor to IPV. However, the cross-sectional design of DHS does not render any causal inferences, merely associations. Such association between controlling behavior and high IPV has been reported previously [24].

Women’s’ and their husband’s educational attainment were both found to be statistically significantly associated with IPV in bivariate but not multivariable model. Previous studies from Pakistan and other countries report lower educational attainment by women and their husbands to be associated with higher associations with IPV [4,5,12,13], while lack of such association has also been reported [28]. Education ostensibly enlightens and bestows better appreciation for fairness, dignity, respect, and human rights of fellow human beings in general and perhaps for one’s spouse in particular; in addition to perhaps better handling of stress and being less inclined to succumb to one’s negative and violent impulses.

Wealth status, involvement in decision-making, and acceptance of IPV were albeit statistically significantly associated with IPV in the bivariate analysis, but not statistically significant in this study using PHDS2017-18 data in the final multivariable model; sharply contrasting with previous studies from Pakistan and other countries [13–16,28,29,32]. Previous study using PDHS2012-13 data [13] found IPV acceptance to be statistically significant in the multivariable model; both PDHSs defined IPV acceptance exactly the same way. However, it used fewer explanatory variables, compared to results reported here e.g. it did not use ‘marital control’ as an explanatory variable. Choice of explanatory variables used in the multivariable model, determines which factors would turn out to be statistically significant after controlling for all the others in the model.

Association of IPV with the use of alcohol by current/former husband, having the knowledge of one’s father physically beat up one’s mother, and marital control are the most consistently reported findings in all DHS surveys. In this study these three attributes, together with number of living children trumped all other attributes in the multivariable model. Previous studies from Pakistan and several other countries testify to these pernicious associations [3,12–14,29–32].

One previous study compared IPV prevalence using PDHS2012-13 and PHS2017-18 data, however no statistical trend analysis was done at the provincial or the capital city levels [33]. Regarding provincial breakdowns, the lifetime prevalence of emotional IPV was highest in the Khyber Pakhtunkhwa (KPK) province, followed by Balochistan, while the lowest prevalence was in the Sindh province; this was true for both surveys. While for physical IPV, the highest prevalence was reported from Balochistan, followed by KPK, and the lowest prevalence was reported from Sindh, in PDHS2017-18. The results from PDHS2012-13 show that highest prevalence of physical IPV was from KPK followed by Balochistan, while the lowest prevalence was reported from Sindh. Since PDHS2012-13 did not inquire about sexual IPV, no comparisons could be made between the two PDHSs. In general, rural areas of provinces reported higher prevalence of emotional and physical IPV, compared with urban areas. The highest prevalence of emotional and physical violence in PDHS2017-18 was reported from rural KPK and rural Balochistan, respectively. In general, prevalence of emotional and physical IPV declined from PDHS2012-13 to PDHS2017-18, at both the cumulative provincial, as well as provincial urban/rural residency status. However, the statistically significant declines were found for emotional IPV in Balochistan and Punjab only; for physical IPV such declines were found for Sindh and KPK only. For urban and rural residency status in each province; emotional IPV declined in rural Punjab, urban Punjab, and urban KPK. While for rural KPK, a statistically significant increase was found for emotional violence, between the two PDHSs. For physical IPV, the only statistically significant change was found for urban Sindh and urban KPK, where the prevalence declined. Cultural shifts, increased awareness about the encumbrance, and more willingness to talk about intimate partner violence between the two surveys might be one plausible explanation for some of the reported discrepancies. Although it’s unlikely that substantial shifts in cultural norms over the relatively short span of five years between the two surveys could account for some of the reported decreases in IPV reporting.

Lack of statistical significance for the urban-rural residency status at the national level using the PHDS2017-18, masked the provincial level as well as provincial urban-rural residency burden and disparities. This augurs the need for more granular analysis to unmask these disparities using sub-nationally representative DHS datasets.

Both surveys i.e. PDHS2012-13 and PDHS2017-18 were restricted to ever married women aged 15–49 and IPV questions were asked in the context of violence perpetrated by the current or the most recent husband. However, the 2018 global estimates [2,3] reported by the WHO and others were 27% for 15–49-year-old ever married and or ever partnered women having experienced physical and/or sexual violence at least once in their lifetime. Using PHDS2017-18 data, the prevalence of ever-married women having ever experienced physical and/or sexual IPV was 23.68%; lower than the global estimate, as well as the WHO Eastern Mediterranean Region’s estimate of 31%.

As both PDHSs only included ever-married women for quantifying the burden of IPV in Pakistan and inquired specifically about IPV experiences perpetrated by either the current or former husband. This constitutes the major limitation of this study, as it obviates the premarital and/or extra-marital IPV experiences of women. Ostensibly, this was done to increase the acceptance and response rates of the surveys by refraining from such culturally hushed intimate relationships. Hence, the prevalence and correlates of IPV are probably an underestimate of the true burden of this public health and human rights menace. IPV is entirely preventable and completely unacceptable criminal behavior, no matter how small the burden. Nonetheless, using the conservative approach of taking the lower bounds of the confidence intervals for the proportions of emotional and physical violence, the burden of IPV is unacceptably higher in some provinces. Moreover, these trends have persisted over the course of five years and two PDHSs, despite showing declines.

Other limitations include the inherent limitations of cross-sectional study design i.e. only associations are deciphered with no causal inferences or temporal associations, and the recall bias. Women aged 50 and older were not included in the PDHSs, as such IPV experiences of these women are not reflected in the various IPV metrics and trends reported in this study. Finally, all IPV measures were self-reported, which could be an underestimate owing to social undesirability, shame in reporting such experiences to interviewers, self-blaming, and especially sociocultural acceptance of such nefarious practices on the part of respondents.

Findings of this study showing IPV burden disaggregated at the provincial urban-rural residency status underscore the critical need for location specific guidelines, policies, and recommendations to effectively address IPV in Pakistan. Wide gulfs between and within provinces in terms of urban/rural residency status, coupled with disturbing acceleration in IPV indices in several subnational regions reflect the disparate norms and practices in the country when it comes to treating one’s spouse by men. Involving men to change this forlorn paradigm is imperative for improving the lot of women in Pakistan.

Use of alcohol, marital control by husband, and having knowledge of one’s father physically beat mother profoundly increased the odds of women reporting IPV. Coupled with the trend of increased number of living children resulting in increased odds of women experiencing IPV, underscore the clear and present need of educational campaigns targeting these correlates to address the IPV menace and attainment of the United Nations Sustainable Development Goals by Pakistan.

Implication of these findings in terms of policy and interventions highlight the imperative impetus for location specific health promotion endeavors, while involving both men and women for changing societal norms would provide the necessary impetus in the long haul to combat the injustices against women. Toothless or meaningless legislation with limited enforcement would only ensure perpetuation of IPV by its perpetrators. This fact was recently underscored and widely reported in the media when a court freed a convicted rapist after reaching an accord to marry his victim [34]. Similar legal edicts would only imperil the status of women in the country and perpetuate continued flagrant violation of their human rights. Meaningfully changing cultural norms and practices, by necessity would be a multifaceted and long-term exercise before positive changes take root in the country. Future iterations of Pakistan DHS, studying IPV metrics at subnational level would enable tracking the progress and finetuning the response strategies and interventions.

In Pakistan, the fight against IPV against women requires a multifaceted approach that tackles both its immediate manifestations and underlying causes. There is a need to actively challenge the societal acceptance of IPV, wife-beating attitudes, and controlling behaviors ingrained in cultural norms. This involves open discussions, awareness campaigns, and promoting healthy relationship models. Breaking the cycle of violence is crucial. Education for both sexes is key, fostering understanding of respectful communication, conflict resolution, and healthy gender roles. This can disrupt the intergenerational transfer of harmful practices that perpetuate IPV. Additionally, empowering women through education and expanded labor market access is vital. Economic independence grants them the freedom to leave abusive situations and build secure lives. Finally, transforming societal norms requires sustained efforts. Engaging community leaders, religious figures, media, and educational institutions is crucial to dismantle patriarchal structures, promote gender equality, and celebrate positive role models. By raising awareness, educating both genders, empowering women, and shifting societal attitudes, the chains of IPV could be broken for creating a future free from violence for Pakistani women.

Future studies in Pakistan need to focus on more granular i.e. the sub-provincial (administrative level of districts) analysis in Pakistan to better capture and understand the nuances of IPV prevalence and its correlates for more focused elimination efforts.

## 5. Conclusion

In Pakistan, the prevalence of physical and/or sexual IPV in the ever-married women perpetrated by the current or most recent husband was lower from both the global as well as the WHO Eastern Mediterranean region. IPV prevalence, in general, was higher in rural areas vis-à-vis urban areas in both PHDSs. Confronting IPV demands a bold move: tackling acceptance and harmful practices through education, empowerment, and social change. Open discussions and education for both genders can rewrite the rules of respect, while economic opportunities offer women a safe path away from abuse. Bridging wide chasms in provincial and urban-rural IPV prevalence disparities in Pakistan underscores the need for location specific interventions through crafting targeted health and social policies to choreograph decrease and ultimately eliminate IPV and human rights burden.
